# Hypoxia regulates glycolysis through the HIF-1α/BMAL1/ALDOC axis to reduce oxaliplatin sensitivity in colorectal cancer

**DOI:** 10.7150/jca.108582

**Published:** 2025-04-28

**Authors:** Jialing Ran, Feifei Li, Lei Zhan, Yue Jin, Qian Dong, Xiaoyan Li, XiaoXi Li, Qian Fei, Jingdong Zhang

**Affiliations:** 1 Cancer Hospital of China Medical University, Cancer Hospital of Dalian University of Technology, Liaoning Cancer Hospital & Institute, PR China.; 2 Shengjing Hospital of China Medical University, PR China.

**Keywords:** hypoxia, HIF-1α, glycolysis, ALDOC, chemotherapy

## Abstract

**Background:** Oxaliplatin (L-OHP) is a first-line chemotherapy agent for advanced colorectal cancer (CRC), but the development of resistance often compromises its efficacy. Tumor hypoxia and metabolic reprogramming are known to influence chemotherapy sensitivity, yet their interrelationship remains inadequately explored.

**Methods:** *In vitro* assays were conducted using human colorectal cancer cell lines (DLD1 and LoVo) under hypoxic conditions induced by cobalt chloride (CoCl2). The expression levels of key proteins involved in the HIF-1α/BMAL1/ALDOC pathway were assessed through Western blotting and quantitative real-time PCR (qPCR). Cell viability, apoptosis, and glycolytic activity were evaluated using CCK-8 assays, flow cytometry, and lactate/ATP measurements.

**Results:** Hypoxia significantly enhanced glycolysis in CRC cells, decreasing sensitivity to L-OHP. The HIF-1α/BMAL1/ALDOC axis was identified as a crucial mediator in this process, with HIF-1α upregulating BMAL1, which increased ALDOC expression. This cascade promoted glycolytic activity and reduced apoptosis in hypoxic conditions. Notably, a positive correlation between HIF-1α and ALDOC expression was confirmed in clinical CRC samples.

**Conclusion:** The findings reveal a novel mechanism by which hypoxia diminishes L-OHP sensitivity in CRC through the HIF-1α/BMAL1/ALDOC pathway. These insights provide potential biomarkers for predicting treatment outcomes and suggest new therapeutic strategies to enhance chemosensitivity in colorectal cancer.

## Introduction

Colorectal cancer (CRC) is a common malignant tumor of the digestive system with a high incidence (1). Oxaliplatin (L-OHP) is a primary chemotherapy drug for CRC, while reduced chemosensitivity is a predominant limiting factor that impedes improved outcomes for patients with advanced CRC. Therefore, it is very important to improve the sensitivity of colorectal cancer to L-OHP treatment to prolong patients' survival.

Metabolic reprogramming within tumor cells is considered as one of the classic hallmarks of cancer 1. The Warburg effect suggests that even in the presence of oxygen, tumor cells switch their metabolic pathway from oxidative phosphorylation to glycolysis, consuming more glucose and producing large amounts of lactate. This process guarantees the synthesis of many cellular elements, the production of energy, and the maintenance of redox homeostasis in cells, which meets the needs of rapid tumor proliferation 2.

The solid tumor mass formed by the rapid proliferation of tumor cells can directly compress the blood vessels around tumor tissue, causing insufficient oxygen supply in the central area of the tumor. Hypoxia can be found within 90% of solid tumors 3, and the hypoxic microenvironment forces cancer cells to undergo genetic and adaptive changes, which promote the malignant transformation of cancer cells 4. Hypoxia-inducible factors (HIFs) play important roles in the above process. HIFs (including HIF-1α, HIF-2α and HIF-3α) regulates oxygen sensing of cells as well as adapts cells to hypoxic microenvironments 5. Under normoxic conditions, HIF-1α is destabilized by regulated degradation 6. However, under hypoxic conditions, HIF-1α forms a stable dimeric structure with HIF-1β that binds to the hypoxia response element (HRE) on DNA to regulate gene expression 7. For example, HIF-1α can directly transcriptionally activate the expression of multiple enzymes (e.g., Aldehyde Carboxylase A, Hexokinase, Lactate Dehydrogenase, etc.) and glucose transporters (e.g., GLUT1) involved in the glycolytic pathway to take up more glucose, accelerate cellular metabolism with energy production, and promote tumor growth 8.

Previous studies have revealed the link between therapeutic resistance and hyperactive glucose metabolism in CRC cells. For example, elevated expression of glucose metabolism-related transporters and key enzymes (e.g. Glucose Transporter1, Hexokinase2) can enhance CRC cell resistance to 5-FU 9-12. Hypoxia is directly related to the glycolysis of tumor cells, and enhanced glycolysis is related to tumors' therapeutic resistance. However, the exact roles of glycolysis regulation by HIF-1α in the therapeutic effects of L-OHP on CRC remain incompletely understood.

Cobalt chloride (CoCl_2_) is a well-established simulant of oxygen deprivation 13. In normoxic conditions, HIF-1α is degraded following the catalysis by prolyl hydroxylases 14. However, CoCl_2_ can stabilize HIF-1α protein levels by inhibiting prolyl hydroxylase expression as well as by activating the MAPK signaling pathways, mimicking hypoxic conditions 15. Preliminary analysis identified potential binding sites for HIF-1α in the promoter regions of BMAL1 and ALDOC, suggesting a regulatory relationship. Furthermore, previous studies have implicated BMAL1 and ALDOC in metabolic reprogramming and tumor progression, particularly under hypoxic conditions. These observations led us to hypothesize that the HIF-1α/BMAL1/ALDOC axis might play a critical role in mediating chemoresistance through glycolytic reprogramming in colorectal cancer 16, 17. In this study, our results suggest that the HIF-1α/BMAL1/ALDOC pathway functions to enhance anaerobic glycolysis in tumor cells, thus reducing the sensitivity of CRC to L-OHP. Our findings provide new evidence for targeting hypoxia in the clinical evaluation and treatment of colorectal cancer patients.

## Material and Methods

### Cell culture

Human colorectal cancer cell lines (DLD1 and LoVo), purchased from the American type culture collection (ATCC, Manassas, VA, USA), were cultured in RPMI-1640 (ScienCell) and MEM media (ScienCell) containing 10% fetal bovine serum (FBS, GIBCO) and 1% penicillin/streptomycin (GIBCO). All cell lines were cultured at 37 °C in a humidified incubator containing 5% CO_2_.

### Western blot

A suitable density of tumor cells was harvested, and all the cells' proteins were extracted using lysis buffer (Beyotime). Protein quantification was performed using a BCA protein assay kit (Beyotime). The protein samples were separated by 10% sodium dodecyl sulfate-polyacrylamide gel electrophoresis (SDS-PAGE) and transferred onto polyvinylidene fluoride (PVDF) membranes (Millipore). Then, the PVDF membranes were blocked with 5% non-fat milk for 1 h at room temperature and incubated with primary antibodies (Table 1) overnight at 4 °C, followed by incubation with fluorescein-conjugated secondary antibodies for one h at room temperature and detected using an enhanced chemiluminescence (ECL) detection kit (Millipore, Burlington, Ma, USA).

### Quantitative real-time PCR (qPCR)

Total RNA of cells was extracted using Trizol (Takara). mRNA was reversely transcribed into complementary DNA (cDNA) using the RT-PCR Kit (Takara) according to manufacturer's instructions. cDNA was used for qPCR experiments with the SYBR Green PCR Master Mix (Takara). The PCR program was set according to the manufacturer's instructions with GAPDH mRNA as an internal control. The relative expression of the gene of interest was calculated by the 2^-ΔΔCt^ method. Primer sequences are shown in Table 2.

### Cell viability assay

According to experimental groups, CRC cells were cultured with conditioned medium containing CoCl2 or L-OHP. Cell survival was assessed using CCK-8 assay (Signatureto laboratories) according to the manufacturer's instructions. Optical density was recorded at 450 nm.

### Clone formation

Cells were cultured for 2-3 weeks with regular observation. After fixation using 4% paraformaldehyde for 15-30 min, crystal violet staining (0.1%) was performed for 30 min. The colonies containing more than 10 cells were counted under a microscope, and the colony formation rate was calculated.

### Apoptosis analysis

Cells were subsequently treated in groups according to the experiment and stained in the dark with 5 μL of FITC-annexin V and 5 μL PI. According to the manufacturer's instructions (BD Pharmingen^TM^), cells were stained for 15 min and analyzed using the flowjo software (version 10.2).

### Adenosine triphosphate (ATP) assay and lactate measurement

CRC cells were treated in groups according to the experiment. ATP and lactate concentrations were determined using an ATP assay kit (Promega Corporation) and a lactate assay kit (Biovision) following the manufacturer's instructions.

### Transfection

The small interfering RNAs were designed (Table 3) and synthesized by Sharp Bio (Guangzhou). CRC cells were transfected with siRNA (50 nM) using Lipofectamine 3000 (Beyotime Biotechnology) for 6 h.

### Immunohistochemistry (IHC)

Firstly, tissue sections were stained to detect the expression of proteins. At 4℃, the sections were combined with the antibody overnight. At 37℃, the sections were bound to the second antibody binding HRP and incubated for 30 minutes. Finally, through DAB dyeing and hematoxylin reversed dyeing (Sigma-Aldridge).

### Clinical samples

33 pathological specimens of colon cancer patients were collected from January 2020 to October 2021 at the Cancer Hospital of Dalian University of Technology (Liaoning, China). The Ethics Committee of Cancer Hospital of China Medical University (20170223) approved this study, which obtained the informed consent of all patients.

### Statistical analysis

All data were independently repeated for 3 times. The results were analyzed and counted by GraphPad Prism 6.0. The unpaired t-test was used to analyze the differences between groups.* p* < 0.05 was considered statistically significant.

## Results

### CoCl_2_-induced hypoxia reduced the sensitivity of CRC cells to L-OHP and enhanced the glycolysis of CRC cells

**Figure 1:**
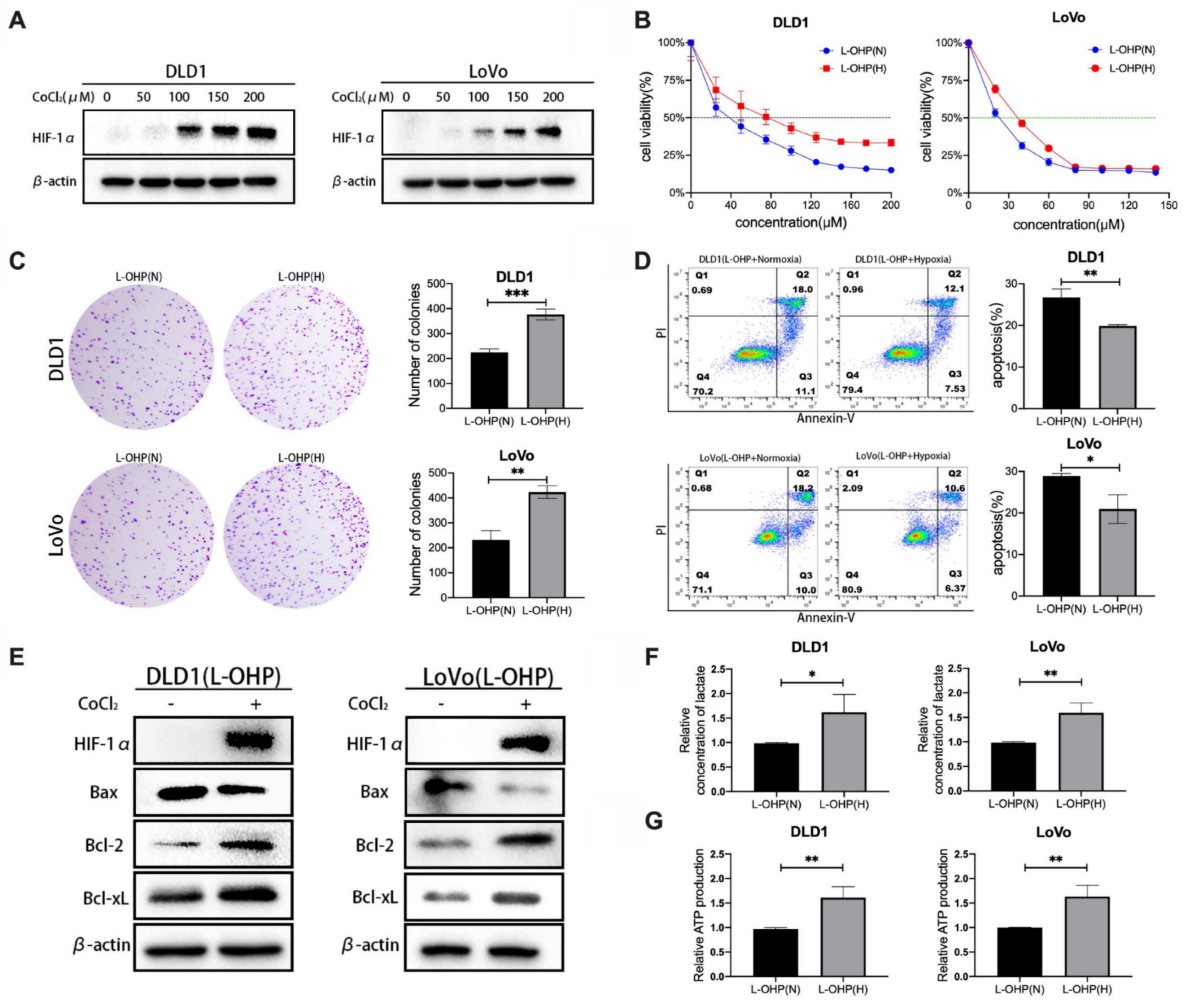
CoCl2-induced hypoxia reduces the sensitivity of CRC cells to L-OHP and enhances glycolysis. (A) Western blot analysis of HIF-1α protein expression under different concentrations of CoCl2. (B) Cell viability assay under normoxic and hypoxic conditions with varying L-OHP concentrations. IC50 values were determined using GraphPad Prism 6.0 software by fitting dose-response curves to a four-parameter logistic model. (C) Clone formation assay results under normoxic and hypoxic conditions with L-OHP treatment. (D-G) Additional assays demonstrating apoptosis, lactate production, and ATP levels. *p < 0.05; **p < 0.01 and * * * p < 0.001).

CoCl_2_ is a common simulant of oxygen deprivation. The 48-h incubation with CoCl_2_ induced the expression of HIF-1α protein in a dose-dependent manner (Figure 1A).

The cell viability of L-OHP-stimulated tumor cells under hypoxic and normoxic conditions was determined. As shown in Figure 1B, L-OHP inhibited the viability of DLD1 and LoVo cell lines under normoxia and hypoxia in a dose-dependent manner, and the experimental results indicated that the cell viability of hypoxic tumor cells under L-OHP stimulation was significantly higher than that of normoxia cells. The half maximal inhibitory concentration (IC50) values of L-OHP treatment for 48 h in normoxia versus hypoxia in the DLD1 cell line were 36.4 μM (95% CI: 33.54 - 39.18 μM) and 70.66 μM (95% CI: 64.27 - 77.04 μM). The IC50 of LoVo cells treated with L-OHP for 48 h in normoxia versus hypoxia were 21.16 μM (95% CI: 18.89 - 23.31 μM) and 34.47 μM (95% CI: 32.14 - 36.77 μM). Clone formation assay was used to compare the proliferative capacity of normoxic versus hypoxic CRC cells under L-OHP treatment to further assess the differences in cell proliferation. The numbers of colony formation of CRC cells under 20 μM of L-OHP treatment were much greater than that of normoxic tumor cells (Figure 1C).

As shown in Figure 1D, the apoptosis rate (calculated as cell percentages with positive annexin-V staining and negative PI staining, as well as cell populations with positive annexin-V staining and positive PI staining) of hypoxic tumor cells treated by L-OHP was significantly lower than that of normoxic cells (*p* < 0.01 for DLD1 and* p* < 0.05 for LoVo). Compared with the normoxia group, the protein expression levels of Bax were significantly downregulated and the protein expression levels of Bcl-2 and Bcl-xL were significantly upregulated in hypoxic tumor cells (Figure 1E). Taken together, these results indicated that hypoxia decreases the sensitivity of tumor cells to L-OHP.

The glycolytic capacity of normoxic and hypoxic tumor cells were examined by lactate generation and ATP generation assays. Lactate and ATP production in hypoxic cells were significantly increased (*p* < 0.05) in L-OHP-treated cells, which reflected the enhanced glycolytic capacity of hypoxic tumor cells (Figure 1F-1G).

### CoCl_2_-induced hypoxia reduces L-OHP sensitivity by modulating the glycolytic capacity of CRC cells

**Figure 2:**
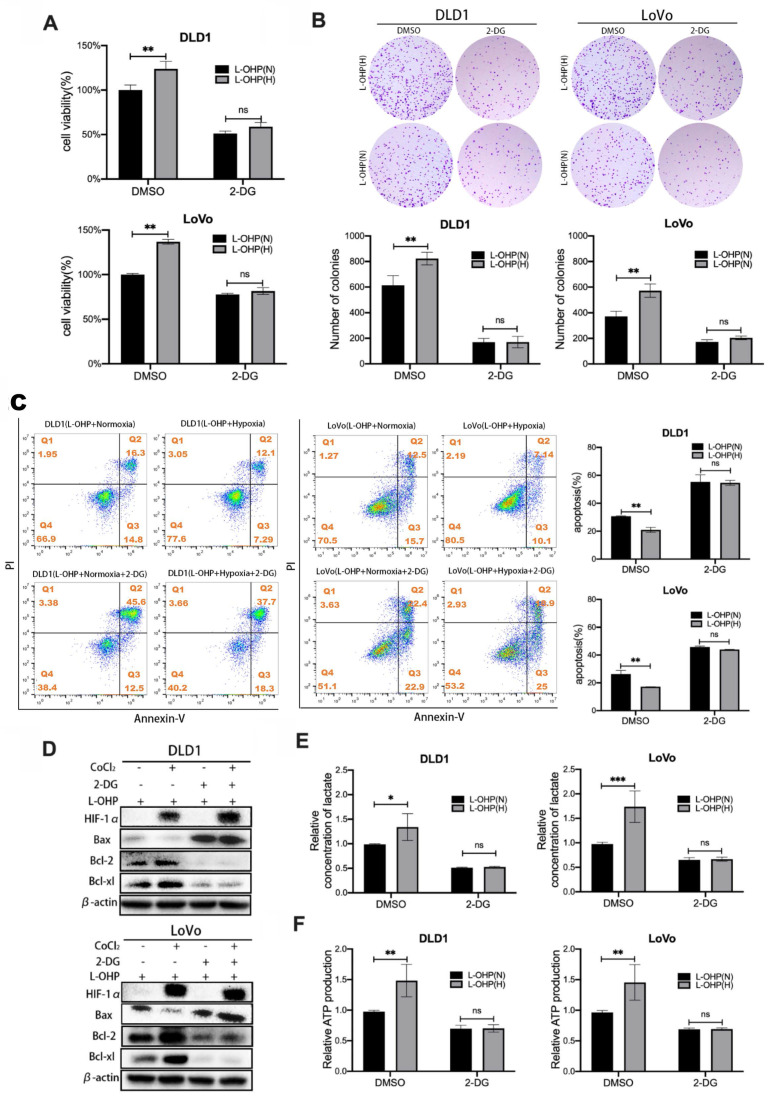
CoCl2-induced hypoxia reduces L-OHP sensitivity by modulating the glycolytic capacity of CRC cells. (A) Cell viability assay detects the cell viability of DLD1 and LoVo cells under normoxia versus hypoxia conditions with L-OHP and 2-DG treatment. (B) Clone formation assay detects the colony numbers of DLD1 and LoVo cells under normoxia or hypoxia conditions with L-OHP and 2-DG treatment. (C) Flow cytometry detects the apoptosis of DLD1 and LoVo cells under normoxia versus hypoxia conditions with L-OHP and 2-DG treatment. (D) Western blot detects the apoptosis-related protein expression in DLD1 and LoVo cells under normoxia versus hypoxia conditions with L-OHP and 2-DG treatment. (E) Lactate measurement assay detects the lactate concentration in DLD1 and LoVo cells under normoxia versus hypoxia conditions with L-OHP and 2-DG treatment. (F) Adenosine triphosphate (ATP) assay detects the ATP production in DLD1 and LoVo cells as indicated. (*p < 0.05, * * p < 0.01, and * * * p < 0.001) (L-OHP (N) represents L-OHP-treated normoxic tumor cells and L-OHP (H) represents L-OHP-treated hypoxic tumor cells).

2-DG is a glucose analog that blocks the initial phase of glycolysis, acting to inhibit glycolysis 18. Cell viability and clone formation assays were used to determine the cell proliferation of L-OHP-treated normoxic and hypoxic tumor cells after 2-DG treatment. As shown in Figure 2A, L-OHP-treated hypoxic tumor cells showed significantly decreased cell viability after 2-DG treatment. Figure 2B also showed that hypoxic tumor cells treated by L-OHP significantly reduced the number of colonies after 2-DG treatment. It was illustrated that L-OHP-treated hypoxic tumor cells subjected to 2-DG treatment inhibited cell proliferation. The apoptosis of tumor cells was detected using flow cytometry (Figure 2C). The experimental results showed that the apoptosis ratio of cells increased after 2-DG treatment in hypoxic tumor cells treated by L-OHP. Apoptosis-related protein expression was detected using Western blot (Figure 2D). L-OHP-treated hypoxic tumor cells showed a significant increase in the expression levels of Bax and a substantial decrease in the expression of anti-apoptotic proteins after 2-DG treatment. These experiments proved that exposure of hypoxic tumor cells to L-OHP and 2-DG treatment promoted the occurrence of apoptosis and enhanced the killing effect of L-OHP on hypoxic cells.

In order to explore the mechanism by which 2-DG affected cell proliferation and apoptosis, lactate and ATP production were measured by lactate measurement kit and ATP content kit in L-OHP-treated normoxic and hypoxic tumor cells after 2-DG treatment. As shown in figure 2E-F, the production of lactate and ATP were obviously decreased after 2-DG treatment in hypoxic tumor cells treated by L-OHP, and the decrease was greater than that in normoxic tumor cells treated by L-OHP. The above experiments illustrated that hypoxic tumor cells treated by L-OHP showed significantly inhibited glycolysis in tumor cells after 2-DG treatment. And the changes were consistent with the apoptosis effects observed in L-OHP-treated hypoxic tumor cells after 2-DG addition, but opposite to the proliferation changes. Therefore, hypoxia possibly decreased L-OHP sensitivity by mediating glycolysis.

### Hypoxia induced by CoCl_2_ reduces L-OHP sensitivity of CRC cells by regulating ALDOC-mediated glycolysis

**Figure 3:**
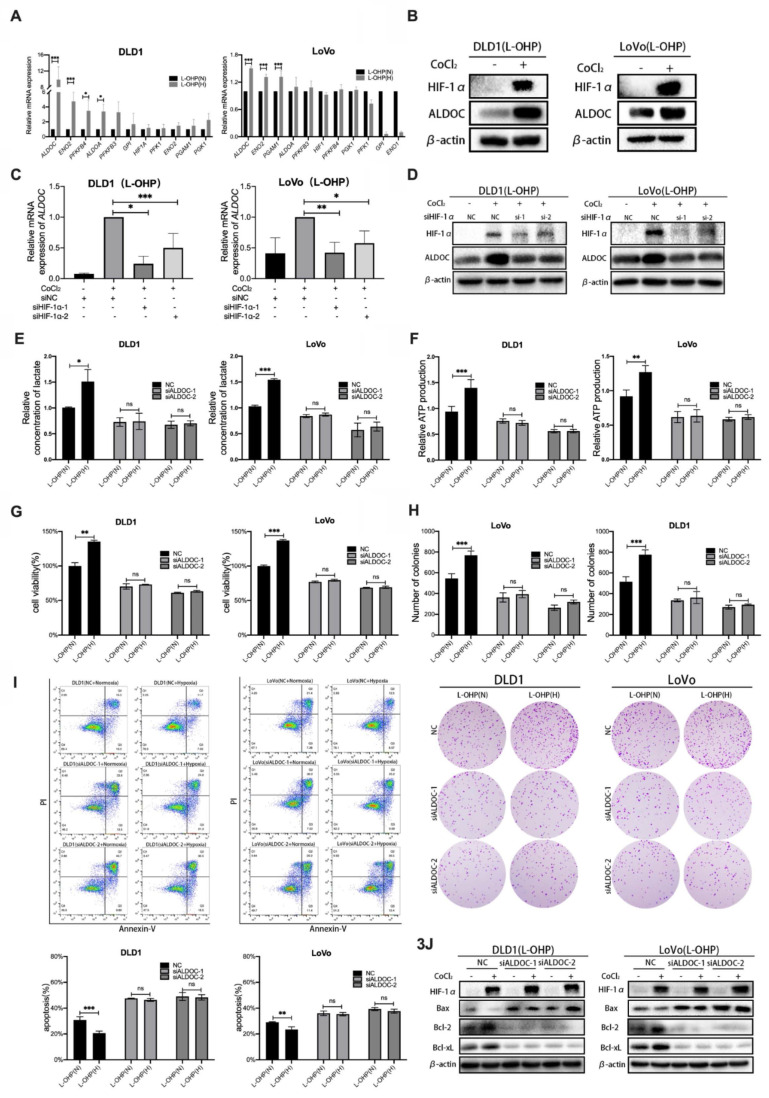
Hypoxia reduces L-OHP sensitivity of CRC cells by regulating ALDOC-mediated glycolysis**.** (A) PCR detects the mRNA expression in DLD1 and LoVo cells under normoxia versus hypoxia with L-OHP treatment. (B) Western blot analyses of the expression of ALDOC protein in DLD1 and LoVo cells under normoxia versus hypoxia with L-OHP treatment. (C) PCR detects the ALDOC mRNA expression in DLD1 and LoVo cells under normoxia versus hypoxia after L-OHP and siHIF-1α treatment. (D) Western blot analyses of the expression of ALDOC protein of DLD1 and LoVo cells under normoxia versus hypoxia after L-OHP and siHIF-1α treatment. (E)Lactate measurement assay detects the lactate concentrations in DLD1 and LoVo cells under normoxia versus hypoxia after L-OHP and siALDOC treatment. (F) Adenosine triphosphate (ATP) assay detects the ATP production of DLD1 and LoVo cells under normoxia versus hypoxia with L-OHP and siALDOC treatment. (G) Cell viability assay detects the cell viability of DLD1 and LoVo cells under normoxia versus hypoxia with L-OHP and siALDOC treatment. (H) Clone formation assay detects the colony numbers of DLD1 and LoVo cells under normoxia versus hypoxia with L-OHP and siALDOC treatment. (I) Flow cytometry detects the apoptosis of DLD1 and LoVo cells under normoxia versus hypoxia with L-OHP and siALDOC treatment. (J) Western blot detects the apoptosis-related protein expression in DLD1 and LoVo cells under normoxia versus hypoxia with L-OHP and siALDOC treatment. (*p < 0.05, * * p < 0.01, and * * * p < 0.001) (L-OHP (N) represents L-OHP-stimulated normoxic tumor cells and L-OHP (H) represents L-OHP-stimulated hypoxic tumor cells).

To explore the molecular mechanism of hypoxia-regulated glycolysis in tumor cells. The qPCR assays were used to screen the expression changes of key enzymes related to glycolysis in normoxic versus hypoxic tumor cells with L-OHP treatment (Figure 3A). The experimental results showed that the expression levels of *ALDOC* mRNA and *ENO2* mRNA were significantly increased in hypoxic cells treated with L-OHP compared with normoxic cells (*p* < 0.001), and the expression difference of *ALDOC* mRNA in the hypoxia and normoxia groups was greater than that of *ENO2* mRNA. The increased expression level of ALDOC protein in hypoxic cells treated with L-OHP was confirmed by Western blot (Figure 3B). The expression levels of both mRNA and protein of *ALDOC* were downregulated (Figure 3C-3D) after the knockdown of HIF-1α, demonstrating the regulatory roles of HIF-1 α on ALDOC expression.

It was aimed to explore whether altering the expression levels of the glycolytic enzyme ALDOC affected glycolysis in CRC cells. The production of lactic acid and ATP after siRNA transfection in L-OHP-treated normoxic or hypoxic CRC cells was detected. Figure 3E-3F showed that the production levels of lactate and ATP were significantly decreased in hypoxic cells treated with L-OHP after ALDOC knockdown. The experimental results illustrated that hypoxic cells treated by L-OHP showed decreased glycolysis of tumor cells after ALDOC knockdown.

It was found that enhancement of glycolysis reduced the sensitivity of tumor cells to L-OHP, while hypoxia enhanced the glycolysis of tumor cells by regulating the expression of ALDOC. However, whether hypoxia decreases L-OHP sensitivity by regulating the expression of ALDOC was unclear. As shown in Figure 3G, L-OHP-treated hypoxic cells showed significantly decreased cell viability after interfering with intracellular ALDOC expression. Figure 3H showed that the numbers of colony formation were decreased considerably in hypoxic cells treated with L-OHP after siALDOC treatment, indicating that cell proliferation was attenuated in hypoxic cells treated with L-OHP after ALDOC knockdown. Flow cytometry results showed that hypoxic cells treated by L-OHP significantly increased the proportion of apoptotic cells after ALDOC knockdown (Figure 3I). Western blot results indicated that L-OHP-treated hypoxic cells exhibited upregulated expression of Bax and decreased expression of Bcl-2 versus Bcl-xL after ALDOC knockdown (Figure 3J). The above experiments illustrated that the expression levels of ALDOC in hypoxic CRC cells treated by L-OHP played an essential role in regulating the sensitivity to L-OHP.

### CoCl_2_-induced hypoxia mediates glycolysis through the HIF-1α/BMAL1/ALDOC pathway to reduce L-OHP sensitivity in colorectal cancer

**Figure 4:**
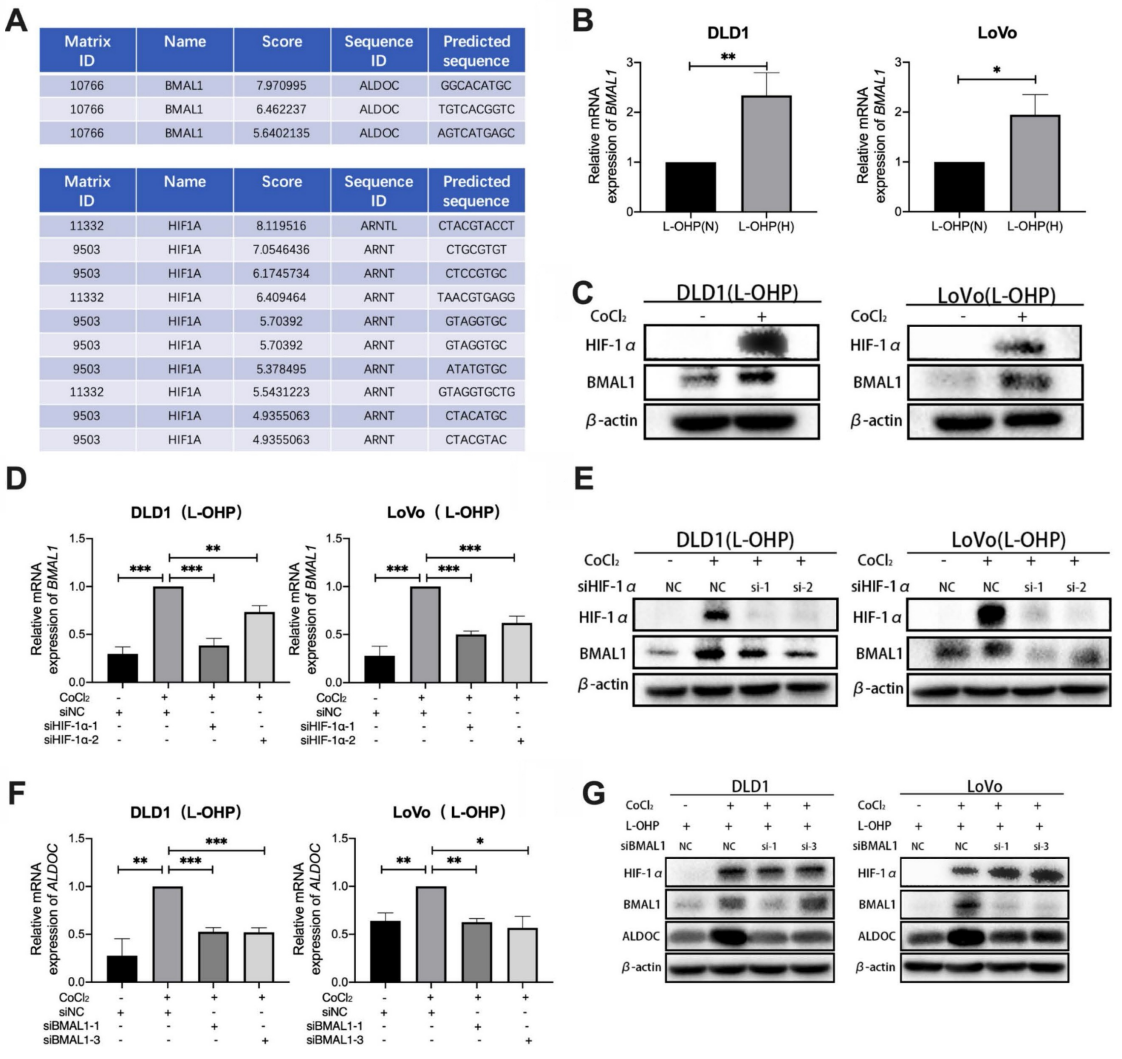
CoCl_2_-induced hypoxia mediates glycolysis through the HIF-1α/BMAL1/ALDOC pathway, thus reducing the sensitivity to L-OHP in colorectal cancer. (A) UCSC and Jaspar online tools predict the binding sites of HIF-1α to *BMAL1* promotor and BMAL1 to *ALDOC* promotor. (B) PCR detects *BMAL1* mRNA expression in DLD1 and LoVo cells under normoxia versus hypoxia with L-OHP treatment. (C) Western blot analyses of the expression of BMAL1 protein in DLD1 and LoVo cells under normoxia versus hypoxia with L-OHP treatment. (D) PCR detects *BMAL1* mRNA expression in DLD1 and LoVo cells under normoxia versus hypoxia conditions with L-OHP and siHIF-1α treatment. (E)Western blot analyses of the expression of BMAL1 protein in DLD1 and LoVo cells under normoxia versus hypoxia with L-OHP and siHIF-1α treatment. (F) PCR detects *ALDOC* mRNA expression in DLD1 and LoVo cells under normoxia versus hypoxia with L-OHP and siBMAL1 treatment. (G) Western blot analyses of the expression of ALDOC protein of DLD1 and LoVo cells under normoxia versus hypoxia with L-OHP and siBMAL1 treatment. (*p < 0.05, * * p < 0.01, and * * * p < 0.001) (L-OHP (N) represents L-OHP-treated normoxic tumor cells and L-OHP (H) represents L-OHP-treated hypoxic tumor cells).

Bioinformatic analysis using the UCSC database showed the presence of binding sites for HIF-1α in the *BMAL1* promoter region (Figure 4A). As shown in Figure 4B-4C, qPCR and Western blot data indicated that BMAL1 mRNA and BMAL1 protein expression were higher in hypoxic tumor cells than in normoxic cells after L-OHP treatment (*p* < 0.05). After treatment with siHIF-1α, L-OHP-treated hypoxic tumor cells exhibited lower *BMAL1* mRNA and protein expression levels than the control group (NC) (Figure 4D-4E). These results illustrated that HIF-1α upregulated BMAL1 expression. UCSC analysis also showed binding sites for BMAL1 in the promoter region of *ALDOC* (Figure 4A). As shown in Figure. 4H-4I, when L-OHP-treated hypoxic tumors were transfected with siBMAL1, the intracellular expression levels of *ALDOC* mRNA and ALDOC protein were lower than those in the NC group, indicating that BMAL1 played a role in regulating ALDOC expression. The above results demonstrated that HIF-1α regulates ALDOC levels by controlling BMAL1 expression.

### The positive correlation between HIF-1α and ALDOC expression was verified in samples of CRC patients

**Figure 5:**
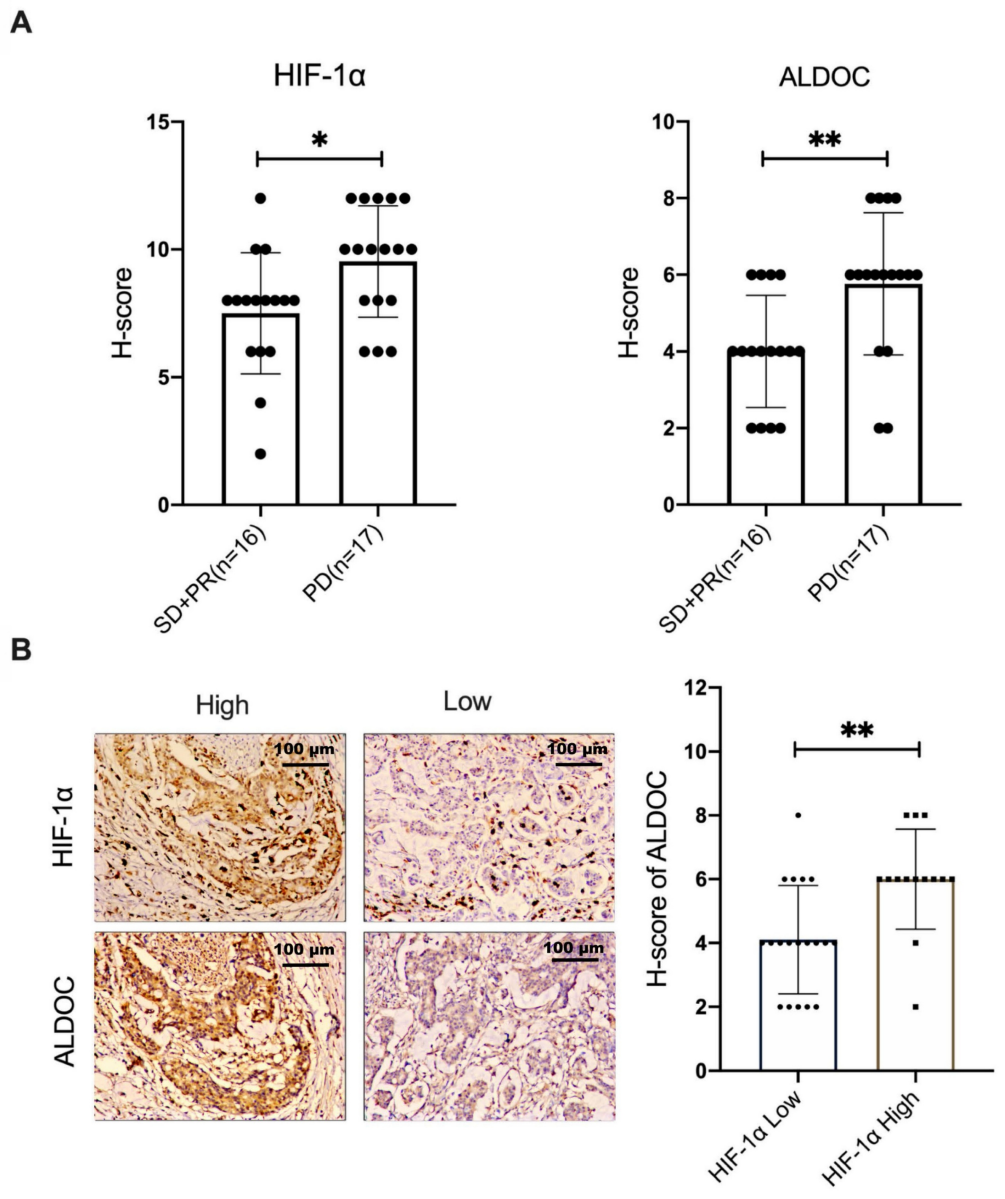
The positive correlation between HIF-1α and ALDOC expression was verified in samples of CRC patients (A) IHC was used to detect the expression of HIF-1α and ALDOC in patients with SD or PR (n=16) and PD (n=17). (B) The correlation between HIF-1α and ALDOC expression was analyzed by IHC. (*p < 0.05, * * p < 0.01, and * * * p < 0.001).

In this study, 33 samples of patients with stage III colon cancer were selected. The selection criteria samples were from the patients who had not received surgery, radiotherapy or chemotherapy before, and all the patients received XELOX chemotherapy based on L-OHP for the first time. The condition was evaluated every 2 cycles. According to Response Evaluation Criteria in Solid Tumors (RECST), samples were divided into two groups. L-OHP-sensitive group (n=16) included stable disease (SD) patient samples and partial response (PR) patient samples. The samples from patients whose response evaluation criteria were progressive disease (PD) were included in the L-OHP-insensitive group (n=17). The protein expression levels of HIF-1α and ALDOC were detected on pathological sections. The results showed that the expression of HIF-1α and ALDOC proteins in the L-OHP-insensitivity group was higher than the L-OHP-sensitivity group (Figure 5A, *p* < 0.05). The localization experiment of protein staining in pathological tissue sections showed that the ALDOC expression was generally high in tissues with high HIF-1α expression. Conversely, the expression of ALDOC was also at a low level (Figure 5B). It was confirmed that there was a positive correlation between HIF-1α and ALDOC (Figure 5B, *p* < 0.05). The above experimental results showed that HIF-1α and ALDOC were highly expressed in the tissues of patients who were not sensitive to L-OHP treatment, and there was a positive correlation between the expressions of HIF-1α and ALDOC, which indicated that ALDOC might be the downstream regulatory target of HIF-1α. HIF-1α and ALDOC were important in enhancing the malignant degree of tumor and reducing the sensitivity of L-OHP chemotherapy. The positive correlation between HIF-1α and ALDOC expression in patient samples suggests their potential as biomarkers for predicting therapeutic response to L-OHP. In the L-OHP-insensitive group, both proteins were highly expressed, indicating their association with chemoresistance. These findings underscore the clinical significance of targeting the HIF-1α/BMAL1/ALDOC axis for overcoming resistance in CRC patients.

## Discussion

Glycolysis is one of the key metabolic pathways for tumor cells. It has been well documented that within many types of cancer, such as liver and gastric cancer, a hypoxic microenvironment will promote tumor proliferation, metastasis and drug resistance by increasing the glycolytic capacity of tumor cells 19-21.

The production of lactate and ATP characterizes glycolysis in cancer cells. Lactate produced by cellular metabolism can be transferred from the cell via the monocarboxylate transporters (MCT), avoiding intracellular lactate accumulation to form a strong acidic environment. Lactate can also render the extracellular microenvironment a weak, acidic state, killing adjacent normal cells and providing conditions for the spreading and metastasis of tumor cells 22. High acidification of the tumor microenvironment has been demonstrated within melanoma to contribute to the immune escape of tumor cells 23. The amount of ATP produced by the glycolytic process guarantees the energy supply for various physiological activities within tumor cells, improving tumors' invasion and metastasis ability 24. This illustrates that glycolysis can promote the malignant transformation of tumors. Our results showed that ATP and lactic acid production in CRC cells induced by hypoxia increased significantly. Therefore, it is a likely important mechanism of tumor progression under hypoxic conditions.

In this study, 2-DG was used to explore the effects of inhibiting glycolysis on cell growth. Our results show that 2-DG effectively blocked lactate and ATP production in L-OHP-treated hypoxic tumor cells. L-OHP-treated hypoxic tumor cells showed suppressed cell viability and colony-forming ability after 2-DG treatment. The increased number of apoptotic cells and enhanced expression of apoptotic proteins illustrate that inhibition of glycolysis in hypoxic cells enhanced the therapeutic effects of L-OHP. Therefore, hypoxia reduced the therapeutic sensitivity of L-OHP by regulating the glycolysis of the tumor.

ALDOC is a member of the aldolase family responsible for the reversible conversion of fructose-1,6-bisphosphate to glyceraldehyde-3-phosphate and dihydroxyacetone phosphate during glycolysis. In gallbladder cancer, disrupting ALDOC protein stability leads to loss of the ability to sense glucose levels inside and outside the cell, thereby activating the AMPK pathway, enhancing tumor glycolysis, and promoting malignant proliferation 25 as well as metastasis 26 of the tumor. In breast cancer, high expression of ALDOC directly enhances glycolysis levels in tumor cells 27. The high expression of ALDOC in ovarian and CRC also suggests poor prognosis of patients 28. Aromatic hydrocarbon receptor nuclear transporter-like (ARNTL), also known as BMAL1, is a circadian clock-related protein. It is well documented that in hepatocellular carcinoma, a hypoxic microenvironment leads to changes in the expression of BMAL1, the core clock gene, thereby promoting tumor progression 29. BMAL1 triggers metabolic reprogramming of cells under inflammatory stimulus conditions 30. The possible mechanism is the interaction between BMAL1 and HIF-1α, leading to the transformation between anaerobic and aerobic glycolysis 31. Previous results showed that knockdown of BMAL1 within the CRC cell line SW480 and SW620 resulted in a decrease in ALDOC expression 32. Therefore, we speculated that HIF-1α might be regulatory in ALDOC expression through BMAL1.

Our transfection experiments verified the regulatory effects of HIF-1α on ALDOC. By measuring lactate and ATP production, we found that glycolysis was inhibited in hypoxic tumor cells treated by L-OHP after siALDOC knockdown, demonstrating HIF-1α exerted a regulatory effect on glycolysis via ALDOC. Proliferation and apoptosis-related experiments confirmed that hypoxic cells showed enhanced therapeutic effects of L-OHP on tumors after siALDOC treatment. Our results were also supported by the UCSC website-based analysis that predicted the transcription factor binding sites on promotor regions of *BMAL1* and *ALDOC*. In a summary, out results concluded the important role of the HIF-1α/BMAL1/ALDOC axis in regulating L-OHP sensitivity in CRC cells.

Epidemiological data show that the mortality rate of CRC is in the third place of malignancy related death worldwide 33, 34. Approximately 20% of patients with CRC already have comorbid metastatic lesions at initial diagnosis, and neoadjuvant therapy provides surgical opportunities for these patients 35. Although surgery is the main method to treat this disease, about 50% patients have recurrence and metastasis after surgery 36. After surgical resection of lesions, chemotherapy becomes an important means to prolong the survival of patients and improve the quality of life of patients. First-line chemotherapy regimens for CRC include XELOX (Oxaliplatin combined with Capecitabine) or FOLFOX (Fluorouracil, Calcium Folinate combined with Oxaliplatin) regimens. Previous studies showed that both regimens improved progression-free survival (PFS) and Overall Survival (OS) in patients with CRC. However, the efficacy was limited 37, 38. A systematic review published in 2020 statistically reviewed published or ongoing clinical trials on L-OHP-based chemotherapy regimens and demonstrated that PFS was only 3.1-7 months 39. The 5-year OS of metastatic CRC is approximately 10% 40. Poor chemosensitivity is a major limiting factor that impedes improved outcomes for patients with advanced CRC. Therefore, it is very important to improve the sensitivity of colorectal cancer to L-OHP treatment to prolong the survival of patients. Our results will shed light on potential therapeutic strategies and treatment for patients who are insensitive to L-OHP. While CoCl2 is widely used as a hypoxia mimic in *in vitro* studies, it may not fully replicate the complexity of physiological hypoxia in tumor microenvironments. CoCl2 stabilizes HIF-1α by inhibiting prolyl hydroxylases but does not account for dynamic oxygen gradients, nutrient deprivation, or stromal interactions present in actual tumors. Future studies using three-dimensional organoid models or *in vivo* systems could provide more comprehensive insights into the role of hypoxia in chemoresistance.

## Figures and Tables

**Table 1:** Manufacturer information of the primary antibody for western blot

Antibody	Manufacturer	Catalog
HIF-1 antibody	*Becton, Dickinson and Company*	610959
ALDOC antibody	*Proteintech Group*	14884-1-AP
Bax antibody	Hangzhou HuaAn Biotechnology	EM1203
Bcl-xL antibody	Hangzhou HuaAn Biotechnology	ET1603-28
Bcl-2 antibody	Hangzhou HuaAn Biotechnology	ET7110-51
BMAL1 antibody	*Proteintech Group*	14268-1-A
β-actin antibody	Cell Signaling Technology	3700S

**Table 2:** qPCR primer sequences

Gene	*Forward primer*	Reverse primer
ENO1	5′- GCCGTGAACGAGAAGTCCTG-3′	5′- ACGCCTGAAGAGACTCGGT-3′
ALDOA	5′- CAGGGACAAATGGCGAGACTA-3′	5′- GGGGTGTGTTCCCCAATCTT-3′
ALDOC	5′- GCCAAATTGGGGTGGAAAACA-3′	5′- TTCACACGGTCATCAGCACTG-3′
PFKFB3	5′- ATTGCGGTTTTCGATGCCAC-3′	5′- GCCACAACTGTAGGGTCGT-3′
PFKFB4	5′- CAACATCGTGCAAGTGAAACTG-3′	5′- GACTCGTAGGAGTTCTCATAGCA-3′
PFK1	5′- AGCTGCCTACAACCTGGTGA -3′	5′- TCCACTCAGAACGGAAGGTGT -3′
PGAM1	5′- GTGCAGAAGAGAGCGATCCG -3′	5′- CGGTTAGACCCCCATAGTGC -3′
GPI	5′- CAAGGACCGCTTCAACCACTT -3′	5′- CCAGGATGGGTGTGTTTGACC -3′
PGK1	5′- GAACAAGGTTAAAGCCGAGCC -3′	5′- GTGGCAGATTGACTCCTACCA -3′
HIF-1α	5′- ATCCATGTGACCATGAGGAAATG-3′	5′- TCGGCTAGTTAGGGTACACTTC-3′
BMAL1	5′- CATTAAGAGGTGCCACCAATCC-3′	5′- TCATTCTGGCTGTAGTTGAGGA-3′
GAPDH	5′- GGAGCGAGATCCCTCCAAAAT -3′	5′- GGCTGTTGTCATACTTCTCATGG -3′

**Table 3:** Target sequences of siRNA

siRNA	Target sequence
si-ALDOC-1	5′- GCAGCACAGTCACTCTACA -3′
si-ALDOC-2	5′- CCTCAAACGTTGTCAGTAT-3′
si-ALDOC-3	5′- GAACGCTGTGCCCAATACA-3′
si-BMAL1-1	5′- GGATGAAGCAACGAACCA-3′
si-BMAL1-2	5′- TCACCAAGATGACATAGGA-3′
si-BMAL1-3	5′- GTCAGAGTTTGTTTGACTA-3′
siHIF-1α-1	5′- GGAAAGGAGAGAAAGCAATT-3′
siHIF-1α-2	5′- AAGCAAAACUCUCAAAACCTT-3′
siHIF-1α-3	5′- GCAAUUCUGGCUCCUACAATT-3′
siNC	5′- ACGUGACACGUUCGGAGAATT-3′

## Abbreviations

CRC: Colorectal Cancer
HIF: Hypoxia-Inducible Factor
HIF-1α: Hypoxia-Inducible Factor 1-alpha
BMAL1: Brain and Muscle Arnt-Like 1
ALDOC: Aldolase C
L-OHP: Oxaliplatin
MTT: A colorimetric assay for measuring cell viability
RT-qPCR: Reverse Transcription Quantitative Polymerase Chain Reaction
ATP: Adenosine Triphosphate
IHC: Immunohistochemistry
FBS: Fetal Bovine Serum
SDS-PAGE: Sodium Dodecyl Sulfate Polyacrylamide Gel Electrophoresis
